# Functional properties of skeletal myotube-derived extracellular vesicles based on microRNA profiles: a comparative analysis with mesenchymal stem cell-derived extracellular vesicles

**DOI:** 10.1038/s41598-026-38076-8

**Published:** 2026-02-05

**Authors:** Yudai Kawamoto, Atomu Yamaguchi, Xiaoqi Ma, Yunfei Fu, Qingcheng Guo, Mikiko Uemura, Hidemi Fujino, Noriaki Maeshige

**Affiliations:** 1 Department of Rehabilitation Science, Kobe University Graduate School of Health Sciences, 7-10-2 Tomogaoka, Kobe, Hyogo 654-0142 Japan; 2 Harvard T.H. Chan School of Public Health, Boston, MA USA

**Keywords:** Extracellular vesicles, Skeletal muscle, Mesenchymal stem cell, microRNA profiling, Enrichment analysis, Cancer, Cell biology, Computational biology and bioinformatics, Molecular biology, Stem cells

## Abstract

Skeletal muscle-derived extracellular vesicles (SkM-EVs) are promising candidates for non-invasive, systemically delivered therapies, but their functional specificity relative to clinically advanced mesenchymal stem cell-derived EVs (MSC-EVs) remains unclear. We reanalyzed public miRNA-seq datasets of SkM-EVs and MSC-EVs and integrated validated miRNA–mRNA interactions to infer pathway-level repression potential of EV miRNA cargo. Two complementary approaches were used: a differential-expression-based relative evaluation with RBiomirGS, and an abundance-weighted absolute evaluation that converts miRNA profiles into gene-level Impact Scores followed by preranked KEGG enrichment. Despite their different formulations, both approaches converged on a shared pattern. SkM-EV miRNAs showed a predicted repression bias in FoxO, TGF-β and ErbB signaling pathways linked to muscle atrophy, metabolic homeostasis and pro-proliferative signaling. By contrast, MSC-EV miRNAs showed a predicted repression bias in immune signaling pathways. These source-dependent pathway signatures provide hypothesis-generating evidence that SkM-EVs may be better suited for muscle-, metabolic-, and cancer-related indications, whereas MSC-EVs may be more appropriate for immunomodulatory indications, pending experimental validation. Our miRNA–target-based framework provides a general strategy to benchmark EV sources at the pathway level directly from miRNA profiles.

**Supplementary Information:**

The online version contains supplementary material available at 10.1038/s41598-026-38076-8.

## Introduction

Skeletal muscle, traditionally known for its primary role in movement, is increasingly recognized as the largest secretory organ in the human body, capable of releasing various factors including extracellular vesicles (EVs)^1^. EVs are 40–160-nm lipid-bilayer nanoparticles released by all cell types, delivering microRNAs (miRNAs) to recipient cells^2^. miRNAs are ~22-nt noncoding RNAs that direct post-transcriptional repression, with conserved targeting predicted for 30–80% of human protein-coding genes^3^. EVs thus function as endocrine and paracrine messengers: by transporting miRNAs, they modulate the phenotype and metabolism of recipient cells^4^. Owing to their high delivery capacity to recipient cells^5^ and the ability to enhance release with ultrasound^6^, skeletal muscle-derived EVs (SkM-EVs) combine inter-organ communication with secretion that can be modulated non-invasively. Previous reports indicate that SkM-EV miRNAs possess anti-inflammatory^7^, pro-myogenic^8^, angiogenic^9^, and osteogenic^10^ properties, and therefore therapeutic applications are highly anticipated.

Clinical translation of EVs is underway, with numerous preclinical and clinical studies in progress. According to the ClinicalTrials.gov database (accessed Oct 2025), 175 EV-based trials have been registered, with the majority (approximately 57%) involving mesenchymal stem cell-derived EVs (MSC-EVs). MSC-EVs are amenable to large-scale manufacturing and can be sourced from bone marrow, adipose tissue, umbilical cord, peripheral blood, and dental pulp^11,12^. Functionally, MSC-EV miRNAs present a wide range of therapeutic functions—including immunomodulation, tissue regeneration (proliferation/migration), anti-apoptotic effects, angiogenesis, anti-fibrotic activity, and neural repair/neuroprotection^13,14^. Although MSC-EVs show high prevalence in clinical trials and broad therapeutic functions, the evaluation of SkM-EVs’ functional properties has lagged behind. It is essential to fully characterize the functional profile of SkM-EVs (absolute evaluation).

EV miRNA profiles are source-specific, because miRNA cargos are actively and selectively sorted in a cell type–dependent manner^15^. SkM-EVs contain high levels of myomiRs (e.g., miR-1, miR-133a/b, miR-206)^16^, whereas MSC-EVs frequently contain broadly acting miRNAs (e.g., let-7 family, miR-143-3p, miR-21a-5p, miR-22-3p)^17,18^. These source-dependent differences in miRNA profiles are likely to translate into distinct functional properties, as each profile will collectively regulate a different set of target genes and, consequently, distinct biological pathways in recipient cells. Given these differences, it is important to compare miRNA profiles of SkM-EVs with those of MSC-EVs, as MSC-EVs represent a major clinical benchmark in EV-based therapies (relative evaluation).

To examine EV functions, prior studies have often adopted a candidate-based approach, focusing on single EV miRNAs^19^. However, this approach is inadequate for evaluating pathway-level regulation, where multiple miRNAs cooperatively target genes within the same biological pathway. Therefore, an abundance-weighted framework based on the EV miRNA profile is required to resolve cooperative, pathway-level regulation.

**Fig. 1:**
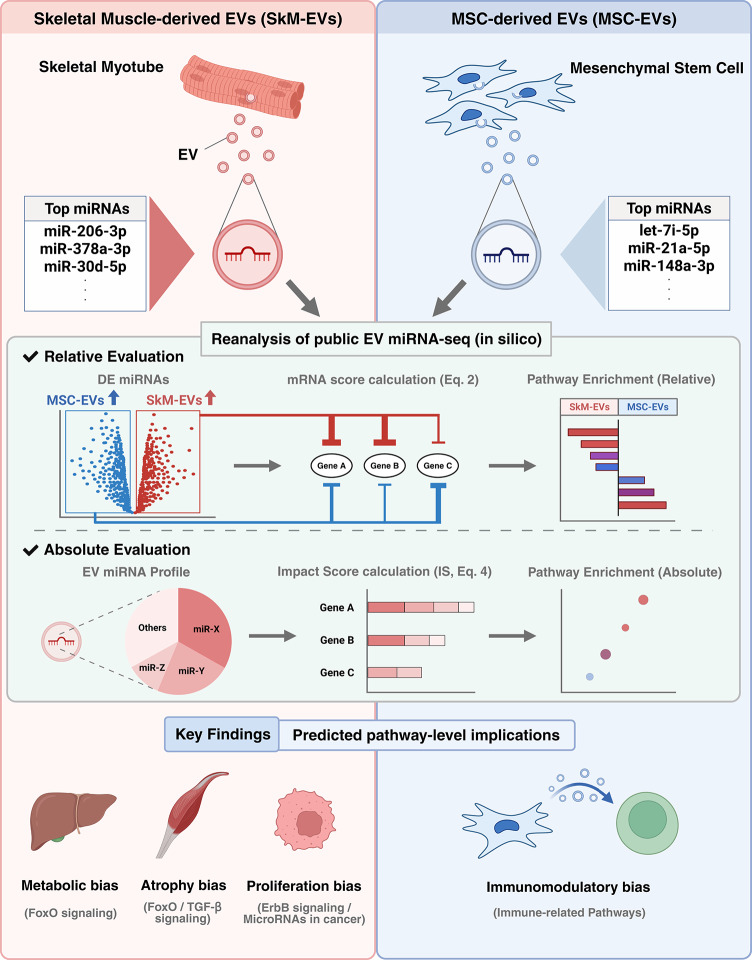
Graphical summary of the study. Created with BioRender.com.

In this study, we mapped normalized miRNA expression to validated targets to compute gene-level Impact Scores (IS) by calculating a weighted sum of all targeting miRNAs per gene, based on their expression levels and miRNA–mRNA interactions. The IS vectors were subjected to preranked Kyoto Encyclopedia of Genes and Genomes (KEGG) pathway analysis to estimate pathway-level effects within each source (absolute evaluation). For the between-source comparison, we combined miRNA differential statistics (fold change and adjusted p-values) with validated miRNA–mRNA interactions to infer, for each pathway, whether predicted repression potential is stronger in SkM-EVs or MSC-EVs (relative evaluation). A graphical summary of the study design and key findings is provided in Fig. 1.

## Results

### miRNA profiles differ between SkM-EVs and MSC-EVs

We reanalyzed publicly available miRNA-seq datasets for SkM-EVs (single-end libraries from C2C12 myotube EVs, PRJNA1044751) and murine bone marrow-derived MSC-EVs (MSC1; PRJNA674943) using a unified pipeline. Quality control and replicate concordance supported selecting MSC1 as the primary MSC comparator (Supplementary Fig. S1). A total of 299 and 360 miRNAs were detected with mean TPM ≥ 5 in SkM-EVs and MSC-EVs, respectively. To assess robustness, we repeated the key analyses using a more permissive detection threshold (mean TPM ≥ 1; Supplementary Fig. S2) and an independent MSC dataset (MSC2; PRJNA523086; Supplementary Figs. S3–S4).

**Fig. 2:**
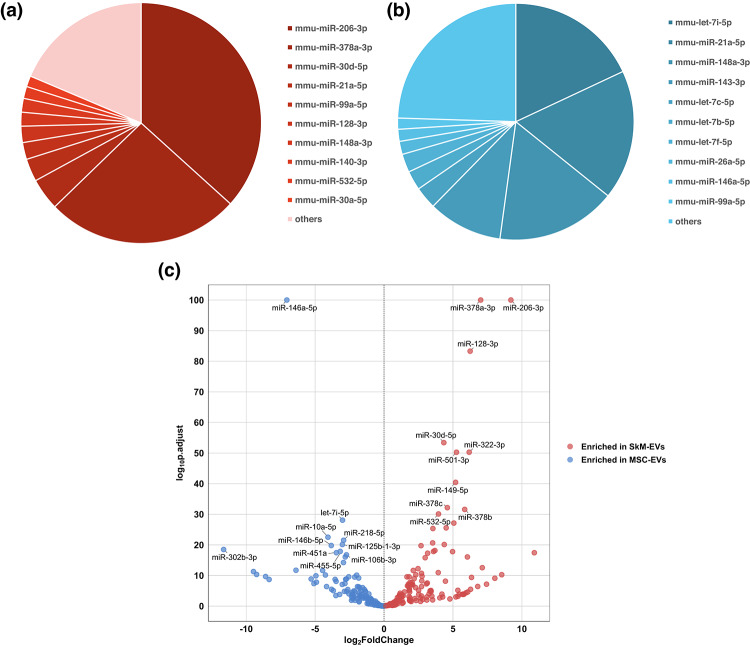
(**a**) Relative abundance of the top 10 expressed miRNAs in SkM-EVs. (**b**) Relative abundance of the top 10 expressed miRNAs in MSC-EVs. In (**a**) and (**b**), each slice denotes the proportion of total normalized reads assigned to an individual miRNA (TPM ≥ 5). (**c**) Volcano plot of differential miRNA statistics between SkM-EVs and MSC-EVs. x-axis shows log2 fold change (SkM-EVs vs. MSC-EVs; positive values indicate enrichment in SkM-EVs), and y-axis shows -log10(adjusted p-value). Points are colored by the enriched source (red: SkM-EVs; blue: MSC-EVs); the 10 most significant miRNAs (smallest adjusted p-values) are labeled.

We quantified the contribution of the most abundant miRNAs within each EV source. In SkM-EVs, a small subset dominated the profile: miR-206-3p and miR-378a-3p together accounted for > 60% of total reads, followed by miR-30d-5p and miR-21a-5p; all remaining miRNAs contributed < 10% individually (Fig. 2a). In MSC-EVs, the distribution was more even, with no single miRNA exceeding 20% of total reads; the top species were let-7 family, miR-21a-5p, miR-148a-3p, and miR-143-3p (Fig. 2b). Exact read counts for all miRNAs and total miRNA reads are provided in Supplementary Table S1. Differential miRNA analysis between SkM-EVs and MSC-EVs was performed to obtain log2 fold change and adjusted p-values for the downstream pathway-level comparison. The volcano plot in Fig. 2c shows the detected miRNAs, highlighting those that differ significantly in abundance between SkM-EVs and MSC-EVs. All detected miRNAs were included in downstream pathway-level analyses without a significance threshold. A complete table of differential miRNA statistics is provided in Supplementary Table S3.

### Pathways with higher predicted repression potential in SkM-EV vs. MSC-EV miRNA profiles (relative evaluation)

**Fig. 3:**
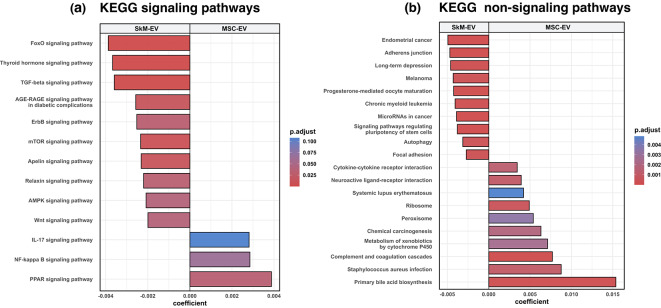
(**a**) KEGG signaling pathways; (**b**) KEGG non-signaling pathways. Bars show the between-source coefficient from RBiomirGS (negative = stronger predicted repression potential in SkM-EVs; positive = stronger predicted repression potential in MSC-EVs). Up to 10 pathways per panel were selected after filtering by adjusted p-values < 0.25; when fewer met the threshold, all qualifying pathways are shown. Color encodes adjusted p within each panel; note that the color scale is different between (**a**) and (**b**). KEGG pathway annotations were obtained from KEGG (www.kegg.jp/kegg/kegg1.html).

To identify pathways differentially impacted by the two EV sources, we analyzed differential miRNA statistics (log2 fold change and adjusted p-values) with RBiomirGS, integrating validated miRNA–mRNA interactions. In KEGG (Fig. 3), SkM-EV miRNAs showed stronger predicted repression potential in the FoxO, TGF-β, ErbB, Wnt, and mTOR signaling pathways, as well as the “MicroRNAs in cancer” pathway. In contrast, MSC-EV miRNA profiles showed stronger predicted repression potential in the NF-κB signaling and “Cytokine–cytokine receptor interaction” pathways. Full ranked lists and statistics are provided in Supplementary Table S4. Note the different color scales between Fig. 3a, b.

### Pathways showing an abundance-weighted predicted repression bias within SkM-EV or MSC-EV miRNA profiles (absolute evaluation)

To further identify pathway-level effects of miRNA cargo within each EV source, gene-level Impact Scores (IS) were computed from miRNA expression (TPM) and validated miRNA–mRNA interactions from TarBase (see Supplementary Table S5). The preranked IS vectors were then subjected to KEGG analysis.

**Fig. 4:**
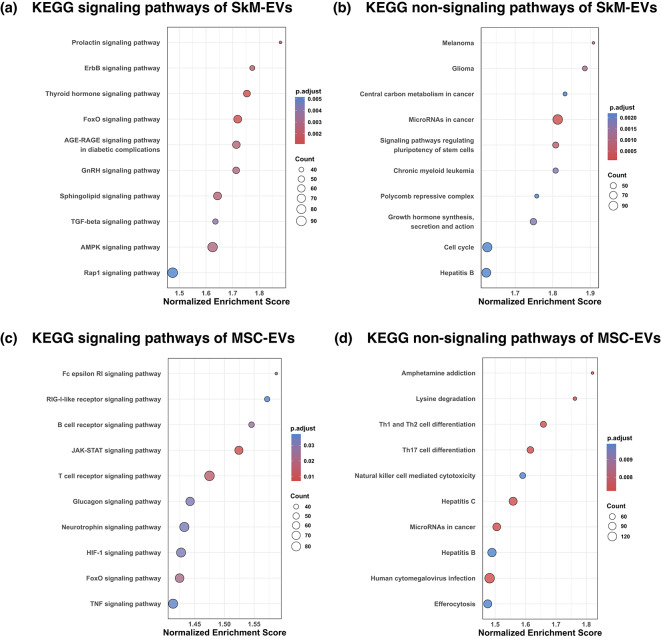
(**a**,** b**) SkM-EVs; (**c**,** d**) MSC-EVs. KEGG pathway analysis was performed on preranked gene-level Impact Scores (IS) derived from the miRNA profiles. (**a**,** c**) show KEGG signaling pathways, and (**b**,** d**) show KEGG non-signaling pathways. The x-axis denotes the normalized enrichment score (NES), which is the enrichment score normalized for gene-set size. Circle size indicates the number of target genes per pathway, and color encodes the adjusted p-values (Benjamini–Hochberg). Up to 10 pathways per panel were displayed, selected by the smallest adjusted p-values. Note that the color scale is different in (**a**)–(**d**). KEGG pathway annotations were obtained from KEGG (www.kegg.jp/kegg/kegg1.html).

In SkM-EVs, KEGG pathway analysis based on miRNA–target interactions indicated a predicted bias toward repression potential of the ErbB, FoxO, AGE-RAGE, TGF-β, and AMPK signaling pathways (Fig. 4a), and “MicroRNAs in cancer” (Fig. 4b). In contrast, MSC-EVs showed a predicted bias toward repression potential of the B cell receptor signaling, T cell receptor signaling pathways (Fig. 4c), and Th17 cell differentiation (Fig. 4d). Complete tables of KEGG analyses are provided in Supplementary Tables S6 and S7. Note the different color scales in Fig. 4a–d.

### Visualization of leading-edge genes across top KEGG pathways showing the strongest predicted repression potential in SkM-EV miRNAs in absolute evaluation

**Fig. 5:**
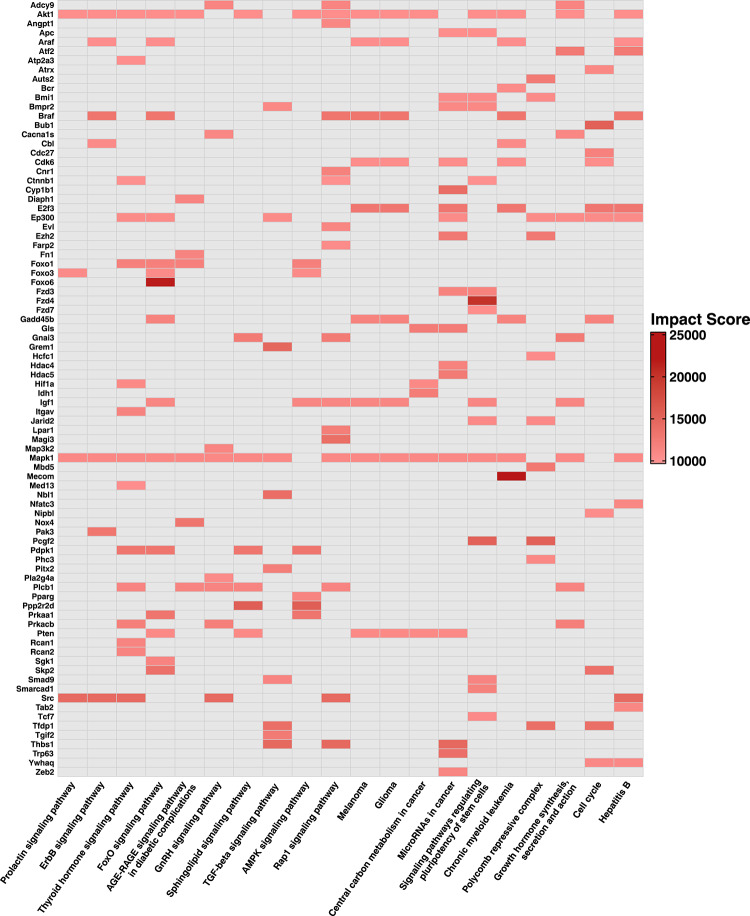
Preranked GSEA was performed on gene-level IS derived from the SkM-EV miRNA profiles. The heatmap summarizes the 20 KEGG pathways shown in Fig. 4a, b (10 signaling and 10 non-signaling) for SkM-EVs. Columns are pathways, ordered by decreasing NES. Rows are the union of leading-edge genes across these pathways. A cell is drawn only when the gene belongs to the pathway’s leading-edge, and the fill encodes the gene’s IS. Gray cells indicate the data were not applicable. Only genes with a maximum IS ≥ 10,000 across the selected pathways are displayed. KEGG pathway annotations were obtained from KEGG (www.kegg.jp/kegg/kegg1.html).

To evaluate the specific genes driving pathway-level predicted repression signature, we used leading-edge analysis within clusterProfiler. Leading-edge genes are a subset of the genes in a particular KEGG pathway that appear prior to the peak score and contribute the most to the NES. The heatmap of leading-edge genes shows miRNA-weighted, gene-level repression signals across the top KEGG pathways biased toward predicted repression potential in SkM-EVs (Fig. 5).

## Discussion

To our knowledge, this is the first study to systematically evaluate SkM-EVs against the clinical benchmark MSC-EVs using a gene–pathway framework weighted by miRNA abundance. By adopting two computational approaches, a relative evaluation based on differences in EV miRNA expression and an absolute evaluation based on the overall abundance of the miRNA profile, we compared the pathway-level regulatory signatures inferred from miRNA profiles of both EV sources. SkM-EVs and MSC-EVs showed clearly different predicted repression patterns across pathways.

These differences are particularly relevant given the unique translational features of SkM-EVs. SkM-EV release in vivo has been reported to be modulated by non-invasive ultrasound^6^, and SkM-EVs show efficient systemic delivery to organs such as the liver and spleen^5^, supporting their potential for clinical application. Therefore, comprehensively understanding the functional specificity of SkM-EVs at the pathway level is essential for maximizing their therapeutic efficacy and rationally differentiating their use from MSC-EVs according to disease pathologies. Importantly, the translational implications discussed here are hypothesis-generating and are derived from computational pathway-level inferences based on EV miRNA profiles. In addition to pathway-level inferences, practical translational considerations—such as biodistribution variability, immunogenicity, EV heterogeneity, and dose consistency^20^—are likely to influence therapeutic performance and should be evaluated in future studies.

In the SkM-EVs vs. MSC-EVs comparison, SkM-EVs showed stronger predicted repression potential in KEGG pathways including FoxO, ErbB, TGF-β, Wnt, mTOR signaling, and “MicroRNAs in cancer” than MSC-EVs (Fig. 3). These pathway sets are implicated in processes such as muscle atrophy (FoxO, TGF-β)^21,22^, glucose metabolism (FoxO, mTOR)^23,24^, fibrosis and keloid scarring (TGF-β, Wnt)^25–27^, and cancer progression (ErbB, Wnt, MicroRNAs in cancer)^28,29^. By contrast, MSC-EVs showed stronger predicted repression potential in NF-κB signaling and “Cytokine–cytokine receptor interaction” pathways, all of which are related to immune responses^30,31^. The orientation of the targeted pathways indicates a predicted repression bias in SkM-EVs toward axes related to metabolic homeostasis and pro-proliferative signaling, while MSC-EVs exhibit a predicted repression bias toward immune-regulatory axes. In the absolute evaluation, SkM-EVs displayed a consistent predicted repression bias in FoxO, ErbB, TGF-β, and “MicroRNAs in cancer” pathways, while MSC-EVs showed a predicted repression bias in immune-regulatory pathways, including B cell receptor signaling, T cell receptor signaling, and Th17 cell differentiation (Fig. 4). Despite the methodological disparities, MSC-EVs have been reported to exert broad immune regulation^32^, and adipose- and umbilical cord-derived MSC-EVs, in addition to bone marrow-derived MSC-EVs, exhibit similar functions^13^. Together, these results suggest that SkM-EV miRNAs are particularly aligned with metabolic homeostasis and anti-proliferative signaling axes, whereas MSC-EVs contribute more to immune regulation. This predicted difference in repression bias between SkM-EVs and MSC-EVs may inform EV source selection for EV-based therapy. These results were supported by sensitivity analyses, including the miRNA detection threshold (mean TPM ≥ 1; Supplementary Fig. S2) and the choice of MSC comparator (MSC2; Supplementary Figs. S3–S4).

In the absolute evaluation of SkM-EVs, the miRNA cargo showed an abundance-weighted predicted repression bias in FoxO signaling, TGF-β signaling, ErbB signaling, and “MicroRNAs in cancer” (Fig. 4a, b). Leading-edge analysis showed that the predicted repression was not uniform across each pathway but concentrated on master regulators (Fig. 5). In FoxO signaling, the leading-edge was dominated by the transcription factors *Foxo1*, *Foxo3*, and *Foxo6*, together with the activity-regulating kinase *Sgk1* and the co-activator *Ep300*. In TGF-β signaling, *Thbs1* was among the principal targets. In ErbB signaling, *Src*, *Mapk1*, and *Akt1* were targeted, and in “MicroRNAs in cancer,” *Cdk6* and *Ezh2* were targeted. The Forkhead box O (FoxO) transcription factors FOXO1, FOXO3, and FOXO6, when dephosphorylated, induce gluconeogenesis in the liver by activating *Pck1* and *G6pc* transcription^23,33^. EP300 promotes gluconeogenesis by increasing FOXO1 protein levels^34^. In addition, SGK1 reduces phosphorylation of FOXO1, thereby maintaining FOXO1 activity and inducing gluconeogenesis^35^. Elevated hepatic gluconeogenesis plays a critical role in the development of obesity and type 2 diabetes and is a major contributor to hyperglycemia in both type 1 and type 2 diabetes. Therefore, suppressing hepatic gluconeogenesis is an important therapeutic strategy. Because SkM-EVs are delivered to the liver at high levels^5^, SkM-EVs may potentially target *Foxo1*, *Foxo3*, *Foxo6*, *Sgk1*, and *Ep300*, suggesting a predicted repression bias within FoxO signaling that could influence gluconeogenesis. Furthermore, dephosphorylated FOXO1 and FOXO3 induce atrophy by driving transcription of Atrogin-1 (*Fbxo32*) and MuRF1 (*Trim63*)^21,36^. Skeletal muscle atrophy is also induced by activation of TGF-β signaling, which acts through activation of SMAD2 and SMAD3^22^. Overexpression of *Thbs1* facilitates TGF-β–mediated muscle atrophy^37^. Thus, the predicted repression bias in FoxO and TGF-β signaling may be consistent with potential modulation of atrophy-related programs. ErbB signaling is strongly associated with cancer cell proliferation, survival, angiogenesis, invasion, and metastasis^38^. Constitutively active ErbB activates multiple cascades including MAPK, SRC, and PI3K-Akt, which mediate ErbB-dependent tumor progression^28^. By centrally targeting leading-edge genes such as *Src*, *Mapk1*, and *Akt1*, SkM-EVs may contribute to repression of ErbB signaling. In the “MicroRNAs in cancer” set, CDK6 and EZH2 are well-known molecules that support cancer cell proliferation and malignant progression and are overexpressed in many cancers^39,40^. CDK6 in “MicroRNAs in cancer” forms a complex with cyclin D to promote G1/S transition^41^ and also enhances tumor angiogenesis^42^. EZH2, the catalytic subunit of PRC2, mediates silencing of tumor suppressor genes through H3K27 trimethylation^43^. Thus, the predicted targeting of these molecules by SkM-EV miRNAs may suggest a potential repression bias against pro-proliferative signaling pathways.

Our study suggests that SkM-EVs show a predicted repression bias in pathways related to metabolic dysregulation, muscle atrophy, and cancer. These pathway-centric findings are consistent with prior reports on the major miRNA cargo of SkM-EVs (Fig. 2a). In metabolic contexts, miR-206-3p, miR-21a-5p, and miR-99a-5p suppress gluconeogenesis^44–46^. In muscle homeostasis, miR-206-3p and miR-378a-3p suppress atrophy and promote myogenic differentiation^47–49^. In cancer contexts, miR-206-3p suppresses renal cell carcinoma^50^, miR-378a-3p suppresses proliferation in prostate cancer^51^, and miR-30d-5p inhibits migration and invasion in esophageal squamous cell carcinoma^52^. These single miRNA functions provide biological plausibility and are consistent with our pathway-level inferences. Thus, our results support the hypothesis that SkM-EVs may exert pathway-level regulatory signatures through coordinated, multi-miRNA repression potential rather than through a single miRNA.

A potential concern in abundance-weighted miRNA analyses is that a small number of highly abundant miRNAs may dominate gene- and pathway-level statistics. This issue is particularly relevant for SkM-EVs, where miR-206-3p and miR-378a-3p together account for > 60% of the total miRNA reads (Fig. 2a). To mitigate undue dominance while retaining the biologically meaningful concept of miRNA cargo load, we incorporated a degree-aware edge weight in the miRNA–mRNA network (Eq. 3). This weighting downscales hub genes with large in-degree and broadly targeting miRNAs with large out-degree, thereby reducing enrichment inflation driven by network topology. In addition, the main pathway signatures were consistent between the relative evaluation (based on differential miRNA statistics) and the absolute evaluation (abundance-weighted Impact Score), supporting that our conclusions are not attributable to a single dominant miRNA.

This study has several limitations that merit discussion. First, this study is a secondary, hypothesis-generating in silico analysis of publicly available miRNA-seq datasets. Therefore, residual batch effects and inter-study heterogeneity cannot be fully excluded, and results should be interpreted with caution. Second, differences in cell culture conditions, EV separation methods, RNA library preparation, and miRNA-seq protocols may influence measured EV miRNA profiles, as highlighted in MISEV guidelines^53^. We also assessed robustness to comparator choice by repeating the key analyses with an independent MSC dataset (MSC2), and the main conclusions were unchanged. Additionally, because EVs also carry non-miRNA cargo, such as proteins, lipids, and other RNA species, our pathway-level inferences are based only on miRNA profiles and do not capture potential contributions from non-miRNA cargo. Furthermore, because the analyzed datasets are murine, species differences may limit direct translation to human biology. Together with the absence of experimental validation, these factors should be considered when interpreting the present computational predictions. Experimental validation of the major pathways identified here will be necessary to confirm these predictions.

In this study, we analyzed data using two computational approaches based on different methods to evaluate pathway-level effects of SkM-EV miRNA cargo. One approach used a differential expression-based relative evaluation compared with MSC-EVs, and the other used a miRNA abundance-weighted absolute evaluation. Our results suggest SkM-EV miRNA profiles exhibit more pronounced predicted repression biases in metabolic, muscle-homeostatic, and cancer-related signaling pathways, whereas MSC-EV miRNA profiles exhibit predicted repression biases preferentially aligned with immune-regulatory pathways.

## Materials and methods

### Data acquisition of EV miRNAs

We acquired publicly available miRNA-seq datasets to obtain SkM-EV and MSC-EV miRNA profiles. Sources consisted of single-end small-RNA libraries from C2C12 myotube-derived EVs (BioProject PRJNA1044751, *n* = 3) and from bone marrow-derived MSC-EVs in two murine studies (MSC1: BioProject PRJNA674943, *n* = 3; MSC2: BioProject PRJNA523086, *n* = 3), all available from the Sequence Read Archive (SRA). Inclusion criteria were Mus musculus origin, EVs collected from C2C12 myotubes (SkM-EV group) or bone marrow-derived MSCs (MSC-EV group), untreated/vehicle culture conditions, and single-end small-RNA libraries.

### Bioinformatics analysis of miRNA-seq data

All miRNA-seq datasets were consistently reanalyzed to ensure comparability between SkM-EVs and MSC-EVs. Quality control was performed using FastQC (v0.12.1)^54^. Sequencing adapters (AGATCGGAAGAGCACACGTCT) were trimmed using Trimmomatic (v0.39)^55^. Reads were aligned to the mouse reference genome mm10 (GRCm38) using STAR (v2.7.11b)^56^. Mature miRNA features were defined from miRBase (v22.1)^57^ and quantified from BAMs with featureCounts (v2.0.6)^58^. Low-abundance miRNAs are prone to stochastic non-detection and increased variability across replicates^59^. In our datasets, miRNAs with mean TPM < 5 frequently showed replicate-level non-detection (TPM = 0 in one or more samples). Therefore, we retained miRNAs with mean TPM ≥ 5 for downstream analyses. We also assessed sensitivity to a more permissive threshold (mean TPM ≥ 1) (Supplementary Fig. S2). The complete TPM values for all miRNAs are provided in Supplementary Table S2.

### Quality control and dataset selection

To characterize inter- and intra-source variability, principal component analysis (PCA) was performed using SkM-EV, MSC1-EV, and MSC2-EV miRNA expression profiles (Supplementary Fig. S1a). PC1 (45.6% variance) separated SkM-EVs from MSC-EVs, with SkM replicates forming a tight cluster on the negative side of PC1. MSC-EV samples occupied the positive side of PC1 but diverged along PC2 (19%). Unsupervised hierarchical clustering of log-scaled, row-z-scored TPM values grouped samples primarily by the cell of origin (Supplementary Fig. S1b). Replicate-level concordance was evaluated across datasets using scatterplot matrix and correlation analysis (Supplementary Fig. S1c). Within each source, biological replicates showed high internal reproducibility, with pairwise Pearson’s r ranging from 0.964–0.976 for SkM, 0.965–0.970 for MSC1, and 0.911–0.945 for MSC2. Sequencing depth was additionally evaluated using total miRNA reads per library (Supplementary Table S1), because low read depth can reduce miRNA profile complexity and the detection of lower-abundance miRNAs in the analysis. Minimum total miRNA read thresholds (≥ 100,000 total miRNA reads) have been adopted in prior quality control frameworks^60^. MSC1 showed consistently higher total miRNA reads across replicates (291,097; 314,833; 165,447), whereas MSC2 showed greater variability and included two of three libraries with < 100,000 total miRNA reads (204,063; 49,596; 40,420). Together with tighter clustering and higher inter-replicate correlations, these quality control metrics supported selecting MSC1 (PRJNA674943) as the primary MSC-EV comparator for the main analyses.

### Validated miRNA–mRNA interactions

Validated miRNA–mRNA interactions were obtained from TarBase v9.0 and deduplicated to retain a single entry per unique miRNA–mRNA pair. This deduplicated interaction set was used consistently as the targeting reference in both the relative evaluation (RBiomirGS) and the absolute evaluation (Impact Score framework).

### Comparative pathway enrichment using RBiomirGS

**Fig. 6:**
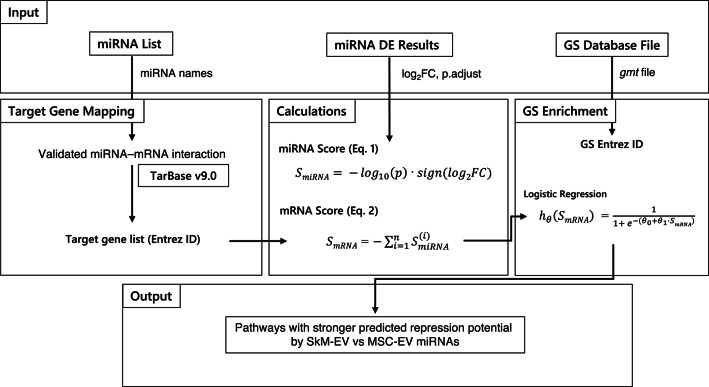
Differential expression statistics for each miRNA between SkM-EVs and MSC-EVs (log2 fold change and adjusted p-values from DESeq2) are first converted into signed miRNA scores (Eq. 1). In the Target Gene Mapping step, these miRNA scores are assigned to their validated mRNA targets, and gene-level scores (Eq. 2) are obtained by aggregating the contributions of all targeting miRNAs. In the GS Enrichment step, RBiomirGS fits a logistic regression model for each KEGG pathway using *S*_*mRNA*_ as predictors to test gene set enrichment. Negative pathway coefficients indicate stronger predicted repression potential in SkM-EV miRNAs, whereas positive coefficients indicate stronger predicted repression potential in MSC-EV miRNAs.

Gene set analysis was performed with the RBiomirGS R package^61^, which implements a logistic regression-based test for enrichment of KEGG pathways^62^. Gene sets were generated to mirror those used by clusterProfiler. KEGG pathways for mouse (mmu) via KEGGREST^63^ were exported to GMT as gene sets for RBiomirGS. All pathway annotations and gene sets were based on the Mus musculus KEGG database (mmu). Gene IDs refer to mouse orthologues, and no human-translated pathway annotation was used. Fig. 6 shows the workflow of this study, adapted from Figure 1 in Zhang et al. (2018)^61^.

For each miRNA, a miRNA score ($\:{S}_{miRNA}$) was computed as.

1 $$\:{S}_{miRNA}=\:-{{log}}_{10}\left(p\right)\:\cdot\:\:sign\left(log2FC\right)$$

where p is the adjusted p-value and FC is the log2 fold change of each miRNA between SkM-EVs and MSC-EVs, estimated using the Bioconductor package DESeq2 (v1.42.1)^64^. Differential expression was performed in DESeq2 using raw integer read counts.

For each mRNA target, an mRNA score ($\:{S}_{mRNA}$) was then calculated by aggregating the scores of all targeting miRNAs:

2 $$\:{S}_{mRNA}=-{\sum\:}_{i=1}^{n}{S}_{miRNA}^{\left(i\right)}$$

 

where *n* is the number of miRNAs targeting that mRNA and $\:{S}_{miRNA}^{\left(i\right)}$ is the score of the *i*-th targeting miRNA. Pathway enrichment was assessed with RBiomirGS using the calculated $\:{S}_{mRNA}$ values. For each KEGG pathway, we fitted a logistic regression (estimated by iteratively reweighted least squares, IWLS) and assessed whether the pathway coefficient differed from zero using a Wald test. Coefficient < 0 indicates stronger predicted repression potential in SkM-EV miRNAs; coefficient > 0 indicates stronger predicted repression potential in MSC-EV miRNAs. Pathways with adjusted p-values < 0.25 were considered significant^61^.

### Impact score for miRNA-weighted repression potential

**Fig. 7:**
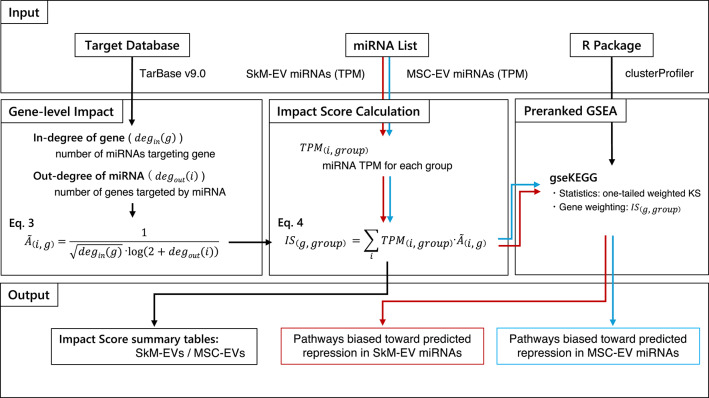
Two inputs are used: a TPM-annotated list of miRNAs detected in SkM-EVs and MSC-EVs, and an experimentally validated miRNA–mRNA interaction database from TarBase v9.0. In the Gene-level Impact module, the bipartite miRNA–mRNA network is restricted to expressed miRNAs, and the in-degree of each gene (number of targeting miRNAs) and out-degree of each miRNA (number of target genes) are computed. These degrees are combined into a degree-aware edge weight *A(i*,* g)* (Eq. 3), which downweights hub genes and broadly targeting miRNAs. In the Impact Score Calculation module, group-level miRNA abundance *TPM(i*,* group)* is multiplied by *A(i*,* g)* and summed over incoming miRNAs to obtain a gene-level Impact Score *IS(g*,* group)* for each group (Eq. 4). In the Preranked GSEA module, genes are ranked by *IS(g*,* group)* and analyzed separately for SkM-EVs and MSC-EVs using preranked GSEA (gseKEGG in clusterProfiler) with a one-tailed weighted Kolmogorov–Smirnov statistic. The outputs are Impact Score summary tables for each group and KEGG pathways showing an abundance-weighted predicted repression potential in each EV source.

As outlined in Fig. 7, two inputs were prepared: (i) a TPM-annotated list of miRNAs detected in SkM-EVs and MSC-EVs, and (ii) an experimentally validated miRNA–mRNA interaction database file from TarBase v9.0.

For each miRNA *i* and group $\:grp\in\:\:\left\{SkM,\:MSC\right\}$, $\:TPM\left(i,group\right)$ denotes the group-level abundance obtained by aggregating sample-level TPM values within the group. Only miRNAs detected in the EV data were retained for downstream analysis. During group aggregation, miRNAs with $\:TPM\left(i,group\right)<5$ in each group were set to zero to reduce low-abundance noise. Using TPM directly retains the contribution of dominant miRNAs and allows each gene to be weighted by the cumulative miRNA targeting load it receives, providing an effect-size-like statistic for preranked pathway analysis.

### Gene-level impact with degree-aware edge weighting

The core idea behind our strategy is to control the heterogeneous many-to-many connectivity between miRNAs and genes. In miRNA analyses, genes with long 3’UTRs, the primary binding sites for miRNAs, tend to accumulate disproportionately many predicted/validated sites and thus exhibit inflated in-degree, which can distort enrichment analyses^65^. Conversely, miRNA promiscuity varies widely; some miRNAs have very large out-degree in both validated and predicted resources^66,67^, allowing them to dominate pathway statistics irrespective of specificity. Using this deduplicated miRNA–mRNA interaction set from TarBase, we retained the native many-to-many structure and computed the in-degree $\:degin\left(g\right)$ (the number of distinct miRNAs targeting gene *g*) and out-degree $\:degout\left(i\right)$ (the number of target genes of miRNA *i*). To prevent high-degree miRNAs/genes from dominating downstream analyses, we applied the degree-aware weighting in Eq. 3.

3 $$\:\stackrel{\sim}{A}\left(i,g\right)=\frac{1}{\sqrt{degin\left(g\right)}\cdot\:{log}\left(2+degout\left(i\right)\right)}$$

 

which applies a concave (square-root) normalization to mitigate hub-gene inflation and a logarithmic penalty to gently compress broadly targeting miRNAs without overly penalizing them. The + 2 term avoids log(0) and prevents undue up-weighting of degree-1 cases.

### Impact score and absolute enrichment (preranked GSEA)

The Impact Score for gene *g* in group *grp* is the TPM-weighted sum over incoming miRNAs:

4 $$\:IS\left(g,group\right)\:={\sum\:}_{i}TPM\left(i,group\right)\cdot\:\stackrel{\sim}{A}\left(i,g\right)$$

 

Each gene was assigned a single value $\:IS\left(g,group\right)$ that served as the preranked statistic for pathway analysis. Gene symbols were mapped to mouse Entrez Gene IDs using org.Mm.eg.db.

Genes were ranked in descending order by $\:IS\left(g,group\right)$. Each group was independently analyzed with preranked GSEA against KEGG mouse (mmu) gene sets using clusterProfiler (v4.10.1)^68^ with a weighted one-tailed Kolmogorov–Smirnov statistic (scoreType="pos”). Pathways with adjusted p-values < 0.05 were considered significant. Larger normalized enrichment scores (NES) indicate that pathway genes are preferentially concentrated near the top of the $\:IS\left(g,group\right)$-ranked list, consistent with higher miRNA-mediated repression potential within the group, whereas NES values near zero indicate little or no enrichment.

### Sensitivity analyses using MSC2 as an alternative comparator

To assess robustness to MSC dataset choice, we repeated both the relative evaluation (RBiomirGS) and the absolute evaluation (Impact Score framework) using MSC2 (PRJNA523086) as an alternative comparator. Results are shown in Supplementary Figs. S3 and S4.

### Statistical analysis

All statistical analyses and data visualization were performed in R (v4.4.2). Principal component analysis (PCA) used prcomp on centered and scaled expression values. Hierarchical clustering was applied to log2(TPM + 1) matrices after row-wise z-scoring, using Euclidean distance and complete linkage. Pairwise similarity between biological replicates was assessed by Pearson’s correlation coefficient (r). For gene set enrichment, pathway p-values from preranked GSEA (one-tailed, weighted KS) and from the logistic regression-based RBiomirGS analyses were adjusted for multiple testing using the Benjamini–Hochberg procedure. All figures were generated with ggplot2 (v3.5.2).

## Supplementary Information

Below is the link to the electronic supplementary material.

Supplementary Material 1

Supplementary Material 2

Supplementary Material 3

Supplementary Material 4

Supplementary Material 5

Supplementary Material 6

Supplementary Material 7

Supplementary Material 8

## Data Availability

This study is a secondary reanalysis of publicly available miRNA-seq datasets. The raw sequencing datasets are available in the NCBI Sequence Read Archive (SRA). SkM-EVs: BioProject PRJNA1044751 (SRA Run accessions: SRR26945942, SRR26945943, SRR26945944). MSC-EVs (MSC1): BioProject PRJNA674943 (SRA Run accessions: SRR13014428, SRR13014429, SRR13014430). MSC-EVs (MSC2): BioProject PRJNA523086 (SRA Run accessions: SRR8599745, SRR8599747, SRR8599748). All processed data supporting the findings of this study are included within the article and its Supplementary Information.

## Abbreviations

EVs: Extracellular vesicles
mRNA: messenger RNA
miRNA: microRNA
SkM-EVs: Skeletal muscle-derived extracellular vesicles
MSC-EVs: Mesenchymal stem cell-derived extracellular vesicles
TPM: Transcripts per million
KEGG: Kyoto Encyclopedia of Genes and Genomes
GSEA: Gene Set Enrichment Analysis
miRNA-seq: miRNA sequencing
IS: Impact Score

## Code availability

The analysis scripts are available from the corresponding author upon reasonable request.
