# Supplementary material for: Functional properties of skeletal myotube-derived extracellular vesicles based on microRNA profiles: a comparative analysis with mesenchymal stem cell-derived extracellular vesicles
Source: Sci Rep. 2026 Feb 5;16:7436. doi: 10.1038/s41598-026-38076-8 (PMC12929730; doi:10.1038/s41598-026-38076-8)
Supplement: Supplementary file 8 — Supplementary Material 8 [file 41598_2026_38076_MOESM8_ESM.docx]

Supplementary Figures

**Functional properties of skeletal myotube-derived extracellular vesicles based on microRNA profiles: a comparative analysis with mesenchymal stem cell-derived extracellular vesicles**

Yudai Kawamoto^1^, Atomu Yamaguchi^1,2^, Xiaoqi Ma^1^, Yunfei Fu^1^, Qingcheng Guo^1^,

Mikiko Uemura^1^, Hidemi Fujino^1^, Noriaki Maeshige^1*^

*^1^Department of Rehabilitation Science, Kobe University Graduate School of Health Sciences, Kobe, Japan*

*^2^Harvard T.H. Chan School of Public Health, Boston, USA*

**Supplementary Figure S1.** (**a**) Principal component analysis (PCA) of normalized miRNA expression (TPM ≥ 5) from skeletal muscle–derived EVs (SkM-EVs) and two independent murine bone-marrow MSC-EV datasets (MSC1, MSC2). (**b**) Unsupervised hierarchical clustering of log₂-scaled, row-z-scored TPM values grouped samples primarily by cell of origin. (**c**) Pairwise scatterplot matrix of miRNA expression (log₂[TPM + 1]) showing replicate-level concordance within each dataset.

**Supplementary Figure S2.** (a, b) SkM-EVs; (c, d) MSC-EVs. KEGG pathway analysis was performed on preranked gene-level Impact Scores (IS) derived from the miRNA profiles filtered using a TPM ≥ 1 threshold. (a, c) show KEGG signaling pathways, and (b, d) show KEGG non-signaling pathways. The x-axis denotes the normalized enrichment score (NES), which is the enrichment score normalized for gene-set size by phenotype permutations. Circle size indicates the number of target genes per pathway, and color encodes the adjusted p-values (Benjamini–Hochberg). Up to 10 pathways per panel were displayed, selected by the smallest adjusted p-values. Note that the color scale is different in (a)–(d). KEGG pathway annotations were obtained from KEGG (www.kegg.jp/kegg/kegg1.html).

**Supplementary Figure S3.** (a) Relative abundance of the top 10 expressed miRNAs in MSC2-EVs. This denotes the proportion of total normalized reads assigned to an individual miRNA (TPM ≥ 5). (b) KEGG signaling pathways; (c) KEGG non-signaling pathways. Bars show the between-source coefficient from RBiomirGS (negative = stronger predicted repression potential in SkM-EVs; positive = stronger predicted repression potential in MSC2-EVs). Up to 10 pathways per panel were selected after filtering by adjusted p-values < 0.25; when fewer met the threshold, all qualifying pathways are shown. Color encodes adjusted p within each panel; note that the color scale is different between (b) and (c). KEGG pathway annotations were obtained from KEGG (www.kegg.jp/kegg/kegg1.html).

**Supplementary Figure S4.** KEGG pathway analysis was performed on preranked gene-level Impact Scores (IS) derived from the MSC2-EV miRNA profiles. (a) shows KEGG signaling pathways, and (b) shows KEGG non-signaling pathways. The x-axis denotes the normalized enrichment score (NES), which is the enrichment score normalized for gene-set size by phenotype permutations. Circle size indicates the number of target genes per pathway, and color encodes the adjusted p-values (Benjamini–Hochberg). Up to 10 pathways per panel were displayed, selected by the smallest adjusted p-values. Note that the color scale is different between (a) and (b). KEGG pathway annotations were obtained from KEGG (www.kegg.jp/kegg/kegg1.html).
